# IL10- and IL35-Secreting MutuDC Lines Act in Cooperation to Inhibit Memory T Cell Activation Through LAG-3 Expression

**DOI:** 10.3389/fimmu.2021.607315

**Published:** 2021-02-17

**Authors:** Marianna M. Koga, Adrien Engel, Matteo Pigni, Christine Lavanchy, Mathias Stevanin, Vanessa Laversenne, Bernard L. Schneider, Hans Acha-Orbea

**Affiliations:** ^1^Department of Biochemistry, Center of Immunity and Infection Lausanne, University of Lausanne, Lausanne, Switzerland; ^2^Brain Mind Institute, École Polytechnique Fédérale de Lausanne, Lausanne, Switzerland; ^3^Bertarelli Platform for Gene Therapy, École Polytechnique Fédérale de Lausanne, Geneva, Switzerland

**Keywords:** dendritic cells, IL-10, IL-35, LAG-3, tolerogenic DCs

## Abstract

Dendritic cells (DCs) are professional antigen-presenting cells involved in the initiation of immune responses. We generated a tolerogenic DC (tolDC) line that constitutively secretes interleukin-10 (IL10-DCs), expressed lower levels of co-stimulatory and MHCII molecules upon stimulation, and induced antigen-specific proliferation of T cells. Vaccination with IL10-DCs combined with another tolDC line that secretes IL-35, reduced antigen-specific local inflammation in a delayed-type hypersensitivity assay independently on regulatory T cell differentiation. In an autoimmune model of rheumatoid arthritis, vaccination with the combined tolDCs after the onset of the disease impaired disease development and promoted recovery of mice. After stable memory was established, the tolDCs promoted CD4 downregulation and induced lymphocyte activation gene 3 (LAG-3) expression in reactivated memory T cells, reducing T cell activation. Taken together, our findings indicate the benefits of combining anti-inflammatory cytokines in an antigen-specific context to treat excessive inflammation when memory is already established.

## Introduction

Dendritic cells (DC) are widely recognized as inducers of adaptive immune responses, modulating the balance between tolerance and immunity. To do so, they rely on the ability to sense the environment upon antigen uptake, migrate, and translate the signals met, not only through upregulation of MHC and co-stimulatory molecules, but also secreting cytokines and inflammatory mediators. Thus, during antigen presentation, DCs are able to activate T cells and direct their fate based on the quality of the signals they expose (1). However, in the context of autoimmune diseases, where exceeding immune activation against self-antigens takes place, it is preferable that the immune response is shifted toward tolerance. Thus, the manipulation of DCs can be advantageous as they represent key players in the development of regulatory responses.

Tolerogenic DCs (tolDCs) are essential for the maintenance of central and peripheral tolerance. They are able to induce clonal T cell deletion, T cell anergy, and regulatory T cell differentiation. DCs can further restrain memory and effector T cell responses due to impaired or sustained antigen presentation, insufficient co-stimulation, and secretion of large amounts of anti-inflammatory mediators (2–5). TolDC differentiation can be favored by many different suppressive factors, like anti-inflammatory cytokines (e.g., TGF-β, IL-10), immunomodulatory drugs (e.g., corticosteroids), vitamin D, and other substances (6, 7). However, the stability of tolDCs and the difficulty in achieving a definitive and efficient induction protocol are issues that still need to be addressed.

The MutuDC1 cell line (CD8α^+^ murine tumor DC line) consists of an immortalized cell line generated through culture of splenic DC tumors from transgenic mice. They were developed and described by our group a few years ago and their functional and phenotypical features resemble the splenic conventional DC1s (cDC1) (8). MutuDC1s are easy to culture *in vitro* and their stability allows further transformation through lentiviral transduction system (9). Therefore, the MutuDC1s represent a great tool to explore the effects caused by the overexpression of immunosuppressive molecules.

We have previously described the generation of a genetically modified MutuDC1 line that constitutively secretes the anti-inflammatory cytokine IL-35 (IL35-DCs). The overexpression of IL-35 in the IL35-DCs was shown to strongly regulate antigen-specific CD4^+^ and CD8^+^ T cell responses *in vitro* and *in vivo*, conferring a tolDC phenotype to the MutuDC1s. In addition, vaccination with IL35-DCs both prevented and ameliorated disease severity on experimental autoimmune encephalitis (EAE), indicating an encouraging approach for treating autoimmune diseases (10). Employing the same approach used to generate the IL35-DC, we have developed a new MutuDC1 cell line that constitutively expresses high amounts of IL-10 (IL10-DC). IL-10 is a potent anti-inflammatory cytokine naturally produced by antigen presenting cells (APCs), B cells, eosinophils, mast cells, and many subsets of CD4^+^ and CD8^+^ T cells upon activation. It has a broad and strong effect on DC function, inhibiting their capacity to produce pro-inflammatory cytokines, upregulating MHC II and co-stimulatory molecules, and impairing their antigen-presenting function (11). The association of IL-10 with other cytokines like the pleiotropic TGF-β was shown to potentiate their individual anti-inflammatory features, leading to the induction of robust regulatory cells in an antigen-specific context (12). This fact indicates that anti-inflammatory cytokines could act in synergy to mediate a tolerogenic response in excessively inflammatory pathologies.

The combination of IL-10 and IL-35 secreted by tumor-infiltrating regulatory T cells was shown to induce the expression of the inhibitory receptors TIM-3, LAG-3, TIGIT, and 2B4, driving intratumoral CD8^+^ T cells to exhaustion (13). LAG-3 is an inhibitory co-receptor involved in controlling excessive activation after persistent antigen exposure. Its expression was observed to play a suppressive role in murine autoimmune disease models of myocarditis, type-1 diabetes, and EAE (14–17), indicating that LAG-3 expression might be modulated by both IL-10 and IL-35 also during CD4^+^ T cell activation.

In this work, we describe our new tolerogenic murine DC line that secretes high amounts of IL-10. We show that when applied in combination with the IL35-DC line, they cooperate to induce antigen-specific tolerance in overly inflamed conditions. Moreover, we show that this cooperation induced the upregulation of LAG-3 expression in memory T cells, dampening the immune response.

## Materials and Methods

### Mice

OT-I/ Rag^−/−^, OT-II, and CD11b^−/−^ mice were bred and kept in our specific pathogen free animal facility. C57BL/6 mice were purchased from Harlan laboratories and kept under the same conditions as mentioned. For all experiments, 8–12-week-old female mice were used, except for the CIA protocol where CD11b^−/−^ sex-matched groups were formed. All experimental procedures were performed in accordance with the Swiss Federal Legislation and approved by the Cantonal Veterinary Office (license number VD.3324).

### Generation of the IL10-Secreting Mutu DC Line (IL10-DC)

The murine tumor DC1 (MutuDC1) line was derived from splenic tumors of transgenic CD11c:SV40LgT C57BL/6 female mouse (8, 18) and the generation and characterization of the IL35-DC line was previously described by Haller et al. (10). For the generation of the IL10-DC line, the Il10 gene was obtained from the cDNA of MutuDC1s stimulated with CpG (1 μM) and amplified by PCR using the following primers 5′-GCCACCAT-GCCTGGCTCAGCACTG-3′ (forward) and 5′-GATCGTCGACTTAGCTTTTCATTTTGAT-CATCAT-3′ (reverse) (synthesized by Invitrogen). The amplified DNA fragments were loaded in 1% Sea Kem GTG agarose gel (Lonza) and purified with the Wizard SV Gel and PCR Clean-up system (Promega) according to the supplier's instructions. The Il10 gene was inserted into the lentiviral vector (pWP-SIN-cPPT-WPRE)-CMV-IRES-GFP and lentiviral particles either empty or containing the IL-10 expression vector (with the GFP reporter) were produced by 293T HEK cells through a second generation transduction system using pMD2G and psPAX2 as packaging vectors. MutuDC1s were stably transduced with the IL-10 lentiviral particles generating the IL10-DC line, or with the empty vector, generating the Mock-DC line. To confirm the transgene expression, protein production was confirmed by FACS (GFP expression and IL-10 expression in GFP^+^ cells) and ELISA. The MutuDC1 lines were cultured in IMDM-Glutamax (GIBCO) supplemented with 10% heat-inactivated fetal bovine serum (FBS Good, PAN-Biotech), 10 mM Hepes (GIBCO), 50 μM β-Mercaptoethanol (GIBCO), 50 U/mL of penicillin, and 50 μg /mL streptomycin (BioConcept) at 37°C in a humidified 5% CO_2_ atmosphere.

### Cell Engineering and Encapsulation for Device Implantation

The murine myoblast C2C12 cell line was obtained from the American Type Culture Collection (ATCC) and cultured with DMEM (GIBCO) supplemented with 10% FBS, penicillin (100 U/mL) and streptomycin (100 μg/mL) (BioConcept). To generate the C2C12 cell lines expressing IL-10 or IL-35, the Il10 gene was inserted into the lentiviral vector pRDI277 (kindly provided by Prof. Richard Iggo, Bergonié Cancer Institute, University of Bordeaux), under the control of the CMV promoter. The Il35 construct (Ebi3 and p35 linked by Gly4Ser) was cloned into the lentiviral expression vector pCDH-CMV-MCS-EF1α-RFP+Puro (System Biosciences). Lentiviral particles were generated by transfection of 293T HEK cells using pVSV-G and psPAX as packaging vectors in serum-free DMEM, in the presence of 1 μg of polyethylenimine/μg DNA. The viral-containing supernatants were collected and filtered (0.22 μM) after 24 h. C2C12 cells were stably transduced with the lentiviral particles and selected for puromycin resistance. Transgene expression was confirmed by FACS and qPCR.

Cell preparation and encapsulation of bioactive cellular implants were previously described (19). Briefly, cells were harvested with Trypsine-EDTA solution and cell suspensions were mixed with PEG gel premix and coagulation factor XIIIa immediately before the loading of 3 × 10^6^ cells (250 μL) into the cell encapsulation devices. The devices were placed on a rocking platform until hydrogel crosslinking was complete, and then sealed with polymerizing medical-grade glue (Loctite, Henkel). The devices were maintained in DMEM for 24 h under cell culture conditions and washed with PBS before subcutaneous (s.c.) implantation in the back of mice. Surgeries were performed under ketamine (100 mg/kg)/xylazine (10 mg/kg) anesthesia and mice recovered in their home cages. Analgesia was provided by a s.c. injection of Buprenorphine (0.5 mg/kg) 24 h after surgery.

### Organ Collection and Processing

Blood was obtained by cardiac puncture, left at room temperature for 30 min and centrifuged for 10 min at 2,000 × g in a refrigerated centrifuge. Sera were collected and stored at −70°C. Draining lymph nodes (DLNs) and spleens were mashed through 40 μm cell strainers. For OT-I, OT-II, and memory assays, T cells were magnetically isolated using the EasySep Mouse CD8^+^ T or CD4^+^ T Cell Isolation Kits (STEMCELL Technologies), following manufacturer's protocols. For the other experimental protocols, cells were treated with ACK lysis buffer (NH4Cl 0.155 M, KHCO3 0.01 M, EDTA 0.1 mM) before they were seeded in culture plates and re-stimulated *ex vivo*.

### *In vitro* OT-I and OT-II Proliferation Assays

10^4^ MutuDCs were seeded in U-botton 96-well plates and pulsed with different concentrations of the ovalbumin peptides SIINFEKL (OVA257-264) (OT-I) or OVA329-337 (OT-II) for 4 h and washed. CD8^+^ or CD4^+^ cells isolated from OT-I/Rag^−/−^ or OT-II mice, respectively, were labeled with 5 μM of the eFluor670 (ThermoFisher) or with Tag-it Violet (Biolegend) proliferation dyes. 10^5^ T cells were then co-cultured with DCs for 72 h.

### Delayed-Type Hypersensitivity (DTH) Assay

C57BL6 mice were immunized against OVA (50 μg – Grade IV, Sigma Aldrich) in Complete Freund's Adjuvant (CFA – InvivoGen). After 7 days, MutuDCs were pulsed with 100 μg/mL of OVA overnight, washed with PBS twice, and 3 × 10^6^ cells were transferred to immunized mice by intraperitoneal (i.p.) injection. When IL10-DCs and IL35-DCs were transferred in combination, they were mixed only a few minutes before the injection, at 1:1 ratio. One week later, mice were challenged with 25 μL of heat-aggregated OVA (20 mg/mL – 500 μg/animal) in one footpad and the same volume of PBS was injected in the contralateral footpad as a control. Footpad thickness was measured with a dial thickness gauge (Mitutoyo) multiple times for 72 h. Blood, lymph nodes and spleen were collected and processed as mentioned above. Total cells were re-stimulated *ex vivo* with 100 μg/mL OVA for 24 h.

### Collagen-Induced Arthritis (CIA)

Chicken collagen type II (CII, Sigma Aldrich) emulsified in CFA (InvivoGen) was prepared as previously described (20) and injected intradermally at the base of tail of the CIA-susceptible CD11b^−/−^ mice. Mice were assessed every day for redness and swelling of limbs or ankle and scored from 1 to 4: (1) erythema and light swelling confined to 1 joint; (2) erythema and mild swelling in one joint or more; (3) erythema and moderate swelling confined to 1 joint; (4) erythema and severe swelling involving multiple joints, joint malformation or ankylosis. No boost of CII was given as mice started scoring positive for the disease as early as 2 weeks after the immunization. In one setup, tolerogenic implants were inserted 1 week after immunization; in another, when around 80% of mice scored at least 1, 5 × 10^6^ MutuDCs pulsed with CII (100 μg/mL) overnight were transferred i.p. When IL10-DCs and IL35-DCs were transferred in combination, they were mixed only a few minutes before the injection, at 1:1 ratio. DC transfer was repeated 2 days later and mice were observed for another 7 days, then euthanized. Blood, lymph nodes and spleen were collected and processed as mentioned above. Total cells from lymph nodes and spleen were re-stimulated with 100 μg/mL of CII *ex vivo* for 24 h.

### T Cell Memory Assays

C57BL6 mice were immunized against OVA (Grade IV, Sigma Aldrich) in CFA (InvivoGen) (50 μg in a total of 100 μL of emulsion per mouse), and boosted after 1 week either with OVA (same concentration) in Incomplete Freund's Adjuvant (IFA, Invitrogen), or with OVA-pulsed MutuDCs (ctrl-DCs). Fourteen days later, spleens were collected and processed as above described. Isolated T cells were labeled with 5 μM of Tag-it Violet proliferation dye (Biolegend). MutuDCs were pulsed overnight with OVA (100 μg/mL), washed twice with PBS, and seeded 96-well plates. DCs and T cells were kept in co-culture for 3 days (10^4^:10^5^ cells per well, respectively).

### ELISA for Cytokine and Antibody Detection

For IL-10 cytokine detection, a specific ELISA kit was used according to manufacturer's instructions (BD Biosciences). To determine OVA- or CII-specific antibodies, plates were coated overnight at 4°C with 20 μg/mL of the appropriate protein in PBS. Plates were washed three times with wash buffer (0.05% Tween-20 in PBS), and blocked with assay diluent (PBS containing 10% heat-inactivated FBS) for 1 h. Mice sera samples were serial diluted in assay diluent and added to plates after three more washes. Following a 2 h incubation at room temperature (RT), plates were washed five times, and anti-IgG1 (clone 2H12B4, Chondrex) or anti-IgG2a (clone 1F10C2, Chondrex) conjugated with peroxidase were used as secondary antibodies. Plates were incubated for 1 h RT and washed seven times. TMB Substrate (Thermo Scientific) was added and plates were left for 30 min RT in the dark. Colorimetric reaction was stopped by the addition of 2 N H_2_SO_4_. Absorbance was acquired at 450 nM in the microplate reader (Ledetect 96, LabExim). Absorbance sample values were considered after subtracting values of wells incubated with fresh serum from naïve mice in the same dilutions or incubated with assay diluent. Data are shown in optical density (OD) units.

### Flow Cytometry Analysis

The fluorochrome-conjugated anti-mouse antibodies used were purchased from Biolegend, ThermoScientific, BD Pharmingen, or R&D Systems, and were specific for: Clec 9A (clone 4D2, PE), MHC-I (Kb) (clone AF6-88.5.5.3, APC), DEC205 (clone 205yekta, PerCP-eFluor710), CD24 (clone M1/69, PerCP-Cy5.5), GR1 (clone RB6-8C5, PerCp-Cy5.5), Langerin (clone eBioL31, PE), CD4 (clone RM4-5, APC, PE-Cy7, PE, or eFluor450), CD11b (clone M1/70, APC), Sirpα (clone P84, APC), MHCII (clone M5/114.15.2, PE), CD11c (clone N418, PeCy7), CD8α (clone 53-6.7, APC-Cy7 or PE-Cy7), B220 (clone Ra3-6B2, eFluor450), CD80 (clone 16-10A1, PE), CD40 (clone 1C10, APC), B7-DC (clone Ry25, PE), PD-L1 (clone 1-111A, PE), CD86 (clone GL-1, AlexaFluor700), MHCII (clone M5, PerCp), CD44 (clone IM7, APC, PE-Cy7, or Pacific Blue), CD62L (clone MEL-14, APC-Cy7, FITC or PE), CD25 (clone PC61.5), TIM-3 (clone B8.2C12, PE-Cy7), LAG-3 (clone C9B7W, APC), FoxP3 (clone FJK-16s, PE or PE-Cy5), EBI3 (clone 355022, APC), P35 (clone IC2191P, PE), IL-10 (clone JES5-16E3, FITC or PE). For intracellular staining, cells were re-stimulated with PMA (10 ng/mL) and ionomycin (500 ng/mL) in the presence of Brefeldin A (10 μg/mL) for 4 h. After extracellular staining, cells were fixed with 3.4% formalin for 15 min at RT and permeabilized with 0.5% saponin buffer for 30 min at 4°C or fixed and permeabilized with the Foxp3/Transcription Factor Staining Buffer Set (ThermoFisher) according to manufacturer's instructions. Cells were analyzed in a LSRII, Canto, or Fortessa flow cytometers (BD), and data processing was done using FACS Diva (BD) and FlowJo (Tree Star). Gates were performed based on Fluorescence Minus One (FMO) controls.

### Statistical Analysis

Results were presented as mean values ± SEM. Statistical analysis were determined by the one-way ANOVA, followed by Tukey's multiple comparison test, or two-tailed Student's *t*-test, using GraphPad Prism software (ns, not significant; ^*^P < 0.05; ^**^P < 0.01; ^***^P < 0.001).

## Results

### IL-10-Secreting DCs: Altered MHC II and Co-stimulatory Molecule Expression at Steady State and Upon Stimulation

**Figure 1:**
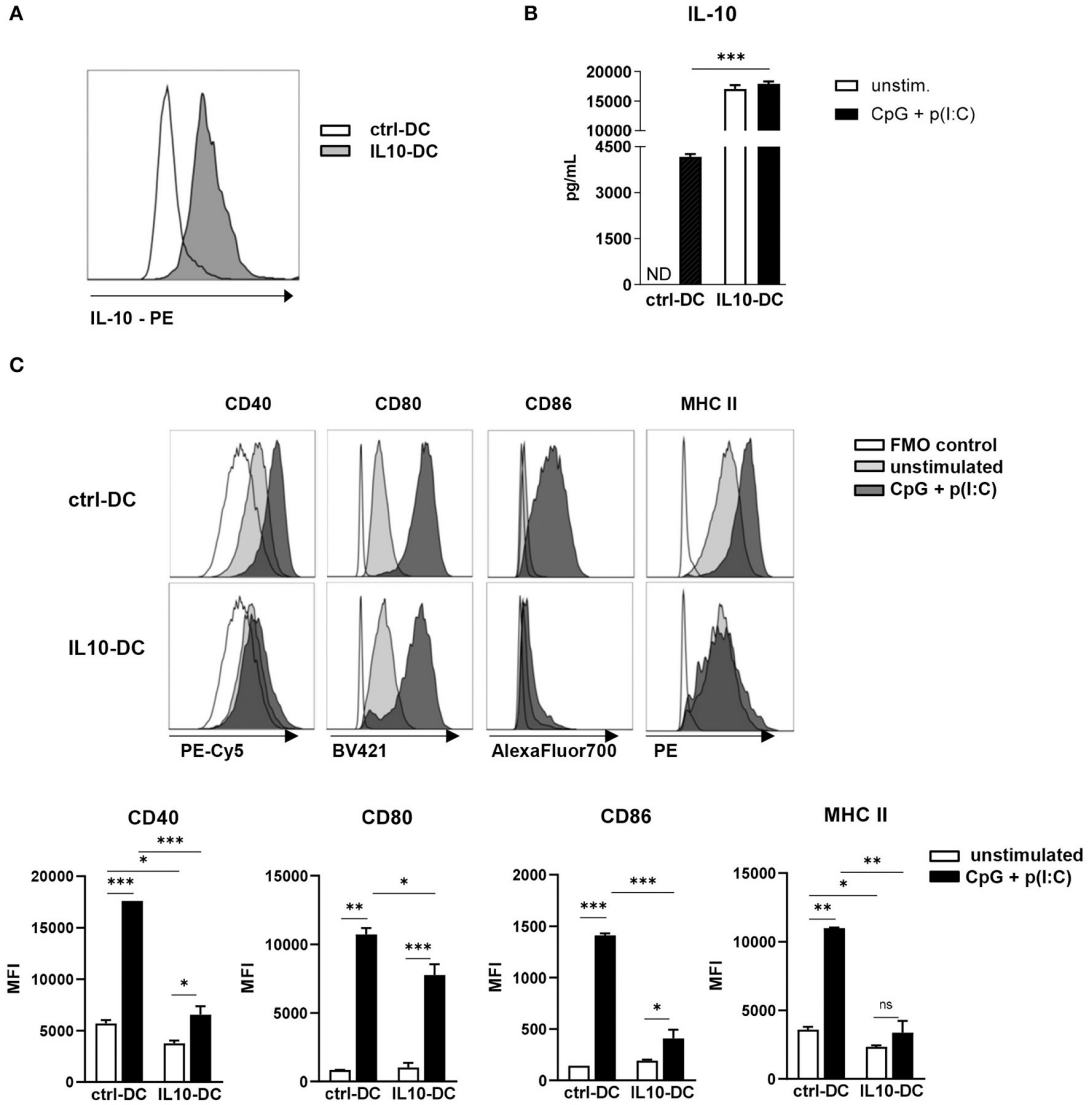
Characterization of the novel IL-10-expressing DC line. 2.5 × 10^5^ control MutuDCs or IL10-DCs or were stimulated with CpG (1 μM) + Poly I:C (5 μg/mL) for 24 h. **(A)** IL-10 expression of control MutuDC (ctrl-DC) and IL10-DC in resting conditions. **(B)** IL-10 secretion assessed in the supernatants of cells cultures. **(C)** Flow cytometric analysis of CD40, CD80, CD86, and MHCII surface expression of ctrl-DC or IL10-DC. Data are expressed in Median Fluorescence Intensity (MFI) of GFP^+^-gated cells. Data are representative of at least three independent experiments. ND, not detected. *P < 0.05; **P < 0.005, ***P < 0.001.

The newly generated IL-10-expressing MutuDC line (referred to as IL10-DC in this work) stably expressed and released IL-10 in resting conditions (Figures 1A,B). In order to test if they kept the MutuDC1 phenotype, we used a panel of surface markers to distinguish DC subsets in IL10-DCs and compared them to untransduced (ctrl-DCs) and mock-transduced MutuDCs. The lentiviral transduction did not affect the expression of the distinctive markers for cDC1s: CD11c, CD8α, DEC205, CD24, and Clec9A, and did not lead to the expression of B220 (specific for pDCs), CD11b, CD4, SIRPα (typically expressed by cDC2s), Gr1 (characteristic for monocytes), or langerin (restricted to dermal DCs) (Supplementary Figure 1). No important modifications in the immature state of the MutuDC1 line were observed after the viral transduction (data not shown).

Upon stimulation with a combination of the TLR ligands CpG (1 μM) and poly(I:C) (5 μg/mL) for 24 h, IL-10 production by IL10-DCs was approximately five times higher than the production of the cytokine by stimulated ctrl-DCs. Contrary to untransduced DCs, the same augmented amount of IL-10 was detected in IL10-DCs cultured in the absence of the stimuli, suggesting that the cells were already producing the cytokine at maximum rate (Figure 1B). In resting conditions, the IL10-DCs expressed lower levels of CD40 and MHC II than control DCs and comparable levels of CD80 and CD86. Upon stimulation with CpG (1 μM) and poly(I:C) (5 μg/mL), IL10-DCs failed to upregulate MHC II expression. CD86 and CD40 were only weakly upregulated upon activation in IL-10 DCs, whereas CD80 expression was only slightly lower in IL-10 DCs than in control DCs (Figure 1C).

### IL10-DCs Induced T Cell Proliferation but Did Not Enhance Treg, Tr1, or iTr35 Differentiation When Combined With IL35-DCs

We next sought to find out if IL10-DCs were able to induce T cell proliferation and what kind of adaptive immune response could be generated in an antigen-specific manner. IL-10 was shown to impair allogeneic and antigen-specific CD8^+^ T cell responses (21). To determine whether IL10-DCs could affect CD8^+^ T cell proliferation, DCs were pulsed with SIINFEKL (OVA_257−264_) for 4 h, washed, and co-cultured with naïve eFluor^670^-labeled OT-I CD8^+^ T cells for 3 days. IL10-DCs induced similar CD8^+^ T cell proliferation compared to control DCs (Supplementary Figure 2A). The expression of perforin (Supplementary Figure 2B) and granzyme B (Supplementary Figure 2C) in CD8^+^ T cells after co-culture was also comparable. Comparable CD4^+^ T cell proliferation was also observed when IL10-DCs or control DCs were pulsed with OT-II peptide (OVA_323−339_) and co-cultured with eFluor^670^-labeled OT-II CD4^+^ T cells (Supplementary Figure 2D). IL10-DCs were similarly able to process full-length ovalbumin and induce antigen-specific CD4^+^ T cell proliferation (Supplementary Figure 2E).

**Figure 2:**
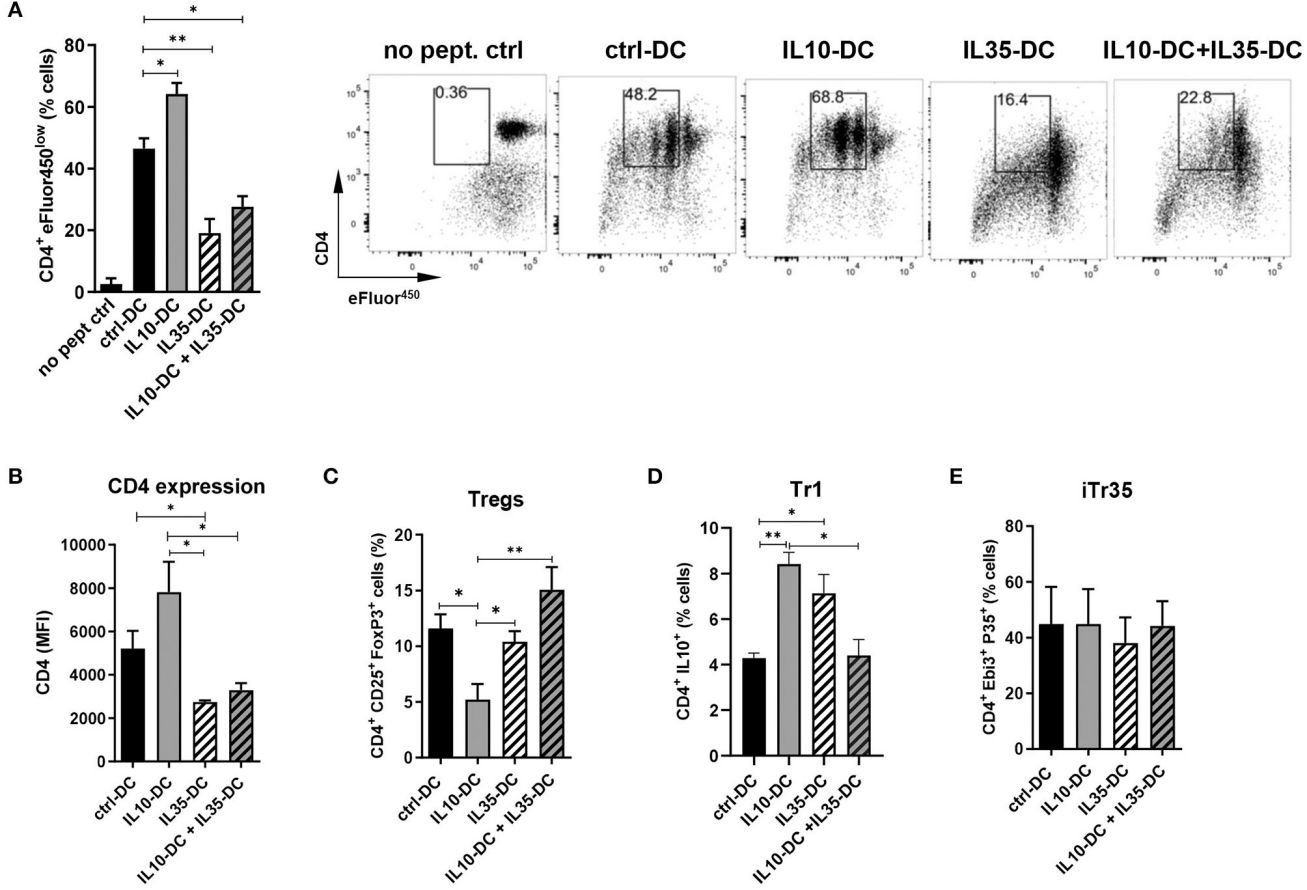
The combination of IL10-DC and IL35-DC did not increase regulatory T cell populations in OVA-specific T cell cultures. 5 × 10^3^ DCs were seeded in 96-well plates and pulsed with OVA_323−339_ peptide for at least 2 h before the addition of 5 × 10^4^ naïve eFluor^450^-labeled OT-II CD4^+^ T cells. IL10-DC and IL35-DC lines, when added together, were mixed in equal dilutions before the seeding. Flow cytometric analysis of **(A)** cell proliferation, **(B)** CD4 expression (given in Median Fluorescence Intensity, MFI, of GFP^−^gated cells), **(C)** Tregs, determined as CD4^+^ CD25^+^ FoxP3^+^ of GFP^−^gated cells, **(D)** Tr1, determined as CD4^+^ IL10^+^ of GFP^−^gated cells, and **(E)** IL35-secreting Tregs (iTr35), determined as CD4^+^ EBI3^+^ P35^+^ of GFP^−^gated cells. Data are representative of at least three independent experiments. *P < 0.05; **P < 0.005.

We have previously thoroughly characterized a high IL-35-producing DC line, also generated from transduced MutuDC1, that expressed low levels of MHC class I and II and failed to upregulate them. Similarly, CD40, CD80, and CD86 was less induced upon stimulation with CpG and poly I:C. When compared to mock-transduced MutuDCs, IL35-DCs induced reduced levels of CD4^+^ and CD8^+^ T cell proliferation (10). Having observed that IL35-DCs could be efficient in changing immunological memory after Th1/Th17 balances were established, we wondered if in combination with the IL10-DC line their tolerogenic features could be potentiated in a synergistic manner. To do that, we tested the combination of our two DC lines in a T cell proliferation assay. We found that the addition of IL10-DCs in the co-culture failed to restore the impaired CD4^+^ T cell proliferation induced by the IL35-DCs (Figure 2A). Interestingly, CD4 expression in T cells appeared reduced when they had been primed by IL35-DCs (Figures 2A,B). The percentages of total induced CD25^+^FoxP3^+^ Tregs were similar, except for a reduced Treg differentiation when the CD4^+^ T cells were co-cultured with IL10-DCs alone (Figure 2C). For Tr1 (CD4^+^ IL10^+^ T cell) differentiation, co-culture with IL10-DCs or IL35-DCs alone was significantly more effective than when the cell lines were combined (Figure 2D). Surprisingly, the regulatory iTr35 population (EBI3^+^ P35^+^ T cells) differentiation was not increased when IL35-DCs were present in the co-cultures (Figure 2E). In order to rule out the effect of the cytokine alone, we polyclonally stimulated T cells in the presence of the supernatant of our DCs in parallel. In this setup, no differences in cell proliferation were observed, but EBI3 MFI was found increased when IL35-DC supernatant was added (data not shown), indicating that the impaired proliferation induced by IL35-DCs is dependent on cell-to-cell contact.

### TolDCs Reduce Antigen-Specific Inflammation *in vivo*

**Figure 3:**
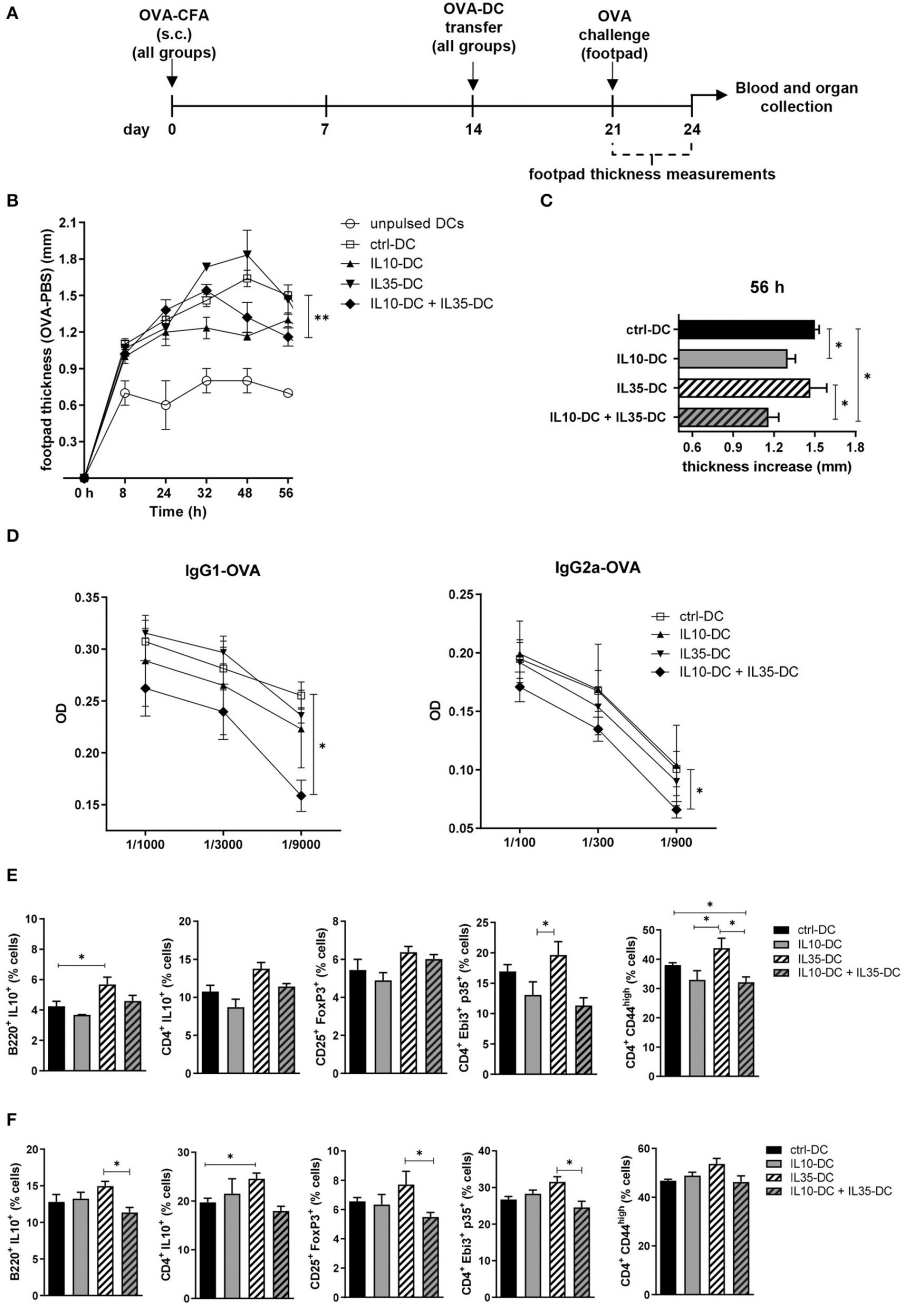
The combination of IL10-DC and IL35-DC ameliorated *in vivo* inflammatory response. Mice were injected s.c. with 50 μg of OVA in Complete Freund's Adjuvant (OVA-CFA) at day 0 and divided in five different groups. At day 14, 3 × 10^6^ OVA-pulsed DCs were injected (via i.p.) and 1 week later they were challenged with a heat-aggregated OVA (20 mg/mL) s.c. injection in the back-right footpad. Same volume of PBS (vehicle) was injected in the contralateral footpad as control. Footpad thickness was repeatedly measured with a micrometer for 56 h before blood and organ collection. **(A)** Schematic timeline of the experimental procedures. **(B)** Footpad thickness measurements of OVA-injected footpad minus PBS-injected footpads. **(C)** Total increase of OVA-injected footpad thickness after challenge. **(D)** OVA-specific IgG1 and IgG2a antibodies in the serum of mice. Cells from the **(E)** inguinal lymph nodes and **(F)** spleen were re-stimulated with OVA (100 μg/mL) in the presence of brefeldin A (10 μg/mL) overnight and T cell populations were assessed by flow cytometry. Data are representative of at least two independent experiments (*n* = 5 mice/group/experiment). *P < 0.05; **P < 0.005.

To observe if the combination of the tolDCs would impact on memory response *in vivo*, we immunized mice with OVA in complete Freund's adjuvant (CFA). Two weeks after the immunization, OVA-pulsed DCs were transferred to immunized mice, which were divided in groups according to the DC line they were going to receive: (1) IL10-DC; (2) IL35-DC; (3) IL10-DC + IL35-DC; (4) ctrl-DC; and finally as a control to see if DCs were boosting the immune response, (5) unpulsed ctrl-DC (Figure 3A). One week after DC transfer, mice were challenged with 25 μL of OVA (20 mg/mL in PBS, 500 μg) in the footpad. The same volume of PBS was injected in the contralateral footpad as a control and footpad thicknesses were measured at multiple time-points. After 48 h of challenge, the mice that were injected with IL10-DCs showed a reduced local inflammatory response compared to the ctrl-DC group. This difference was still detected after 56 h, when the footpads of mice from the IL10-DC + IL35-DC group also showed decreased swelling compared to the ctrl-DC and the IL35-DC groups (Figures 3B,C). Also, OVA-specific IgG1 and IgG2a antibodies were found decreased in the sera of mice from the IL10-DC + IL35-DC group (Figure 3D). We then re-stimulated total cells from the inguinal draining lymph nodes (Figure 3E) or spleen (Figure 3F) with OVA *ex vivo* and we observed a slight increase of the Breg (B220^+^ IL10^+^ cells – lymph nodes) and Tr1 (CD4^+^ IL10^+^ – spleen) cell populations in the IL35-DC group compared to the ctrl-DC group. Surprisingly, we did not find any differences in the frequency of Bregs or Tr1 in the IL10-DC group or when the two cell lines were combined. In fact, the IL10-DC + IL35-DC group showed reduced numbers in the regulatory cell populations compared to the IL35-DC group, but also decreased numbers of CD4^+^CD44^high^ cells in the DLNs, indicating impaired T cell activation.

### Prevention and Treatment of Established Collagen-Induced Arthritis (CIA)

**Figure 4:**
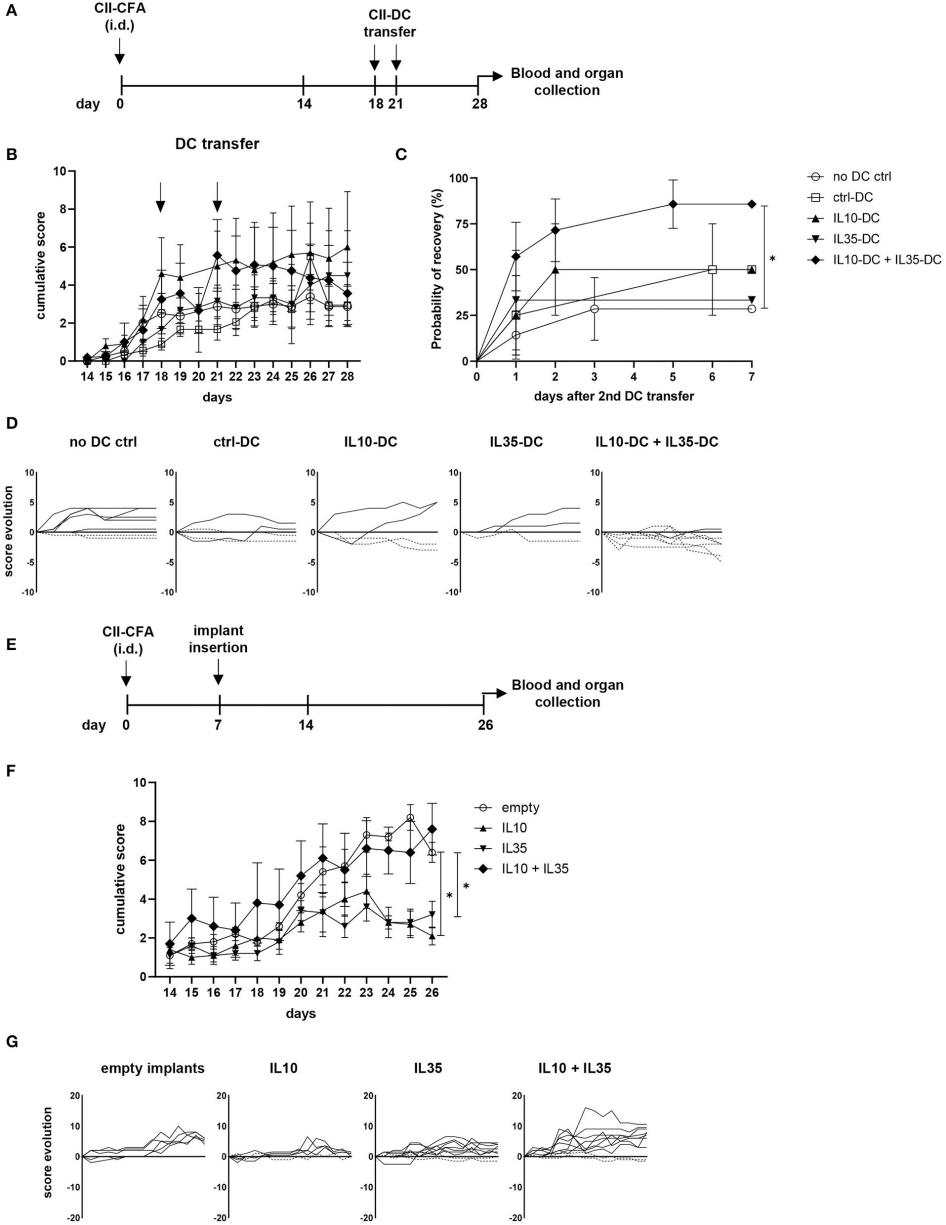
IL10-DC + IL35-DC vaccination reduced established CIA clinical scores. CIA was induced with an i.d. injection at the base of the tail of 100 μg of chicken collagen II emulsified in Complete Freund's Adjuvant (5 mg/mL) (CII-CFA). At day 18, when 80% of mice were showing at least mild scores of the disease, mice were vaccinated with 5 × 10^6^ CII-pulsed DCs (CII-DC). Treatment was repeated 2 days later. Arthritis severity was evaluated daily and each paw was individually scored for erythema, swelling, and ankylosis. **(A)** Schematic timeline of the experimental procedures. **(B)** Cumulative arthritis scores; CII-DC transfers are indicated by the arrows. **(C)** Probability of recovery evaluation of mice after the second DC transfer based on cumulative score reduction: each mouse was analyzed for having its scores reduced or not. **(D)** Individual score evolution of sick mice after the second DC transfer: each line represents the increase (full lines) or decrease (dashed lines) of the cumulative score (*y* axis) of one individual during days 21 to 28 (*x* axis). In a second approach, 1 week after immunization, five groups of mice were implanted with bioactive cellular implants secreting IL-10, IL-35, IL-10, and IL-35, or empty implants. **(E)** Schematic timeline of the experimental procedures. **(F)** Cumulative arthritis scores. **(G)** Individual score evolution of sick mice 1 week after the implantation of device: each line represents the increase (full lines) or decrease (dashed lines) of the cumulative score (*y* axis) of one individual during days 14 to 26 (*x* axis). Data are representative of at least two independent experiments (*n* = 3–10 mice/group/experiment). *P < 0.05.

Rheumatoid arthritis (RA) is an autoimmune disease generally characterized by exacerbated Th1 and Th17 responses, which cause and sustain joint inflammation. Systemic delivery of anti-inflammatory cytokines, such as IL-10 and IL-35 has been shown to prevent the onset of collagen-induced arthritis (CIA) and to treat its symptoms, but the short half-life of some cytokines require constant delivery and high concentrations of the substance, which can cause secondary effects associated with excessive anti-inflammatory response (22, 23). More target-specific approaches employing peptides or cell transfer have also been tested and showed promising results (24). Taking advantage of our tolerogenic cytokine-secreting DC lines, we tested the therapeutic effect of IL-10 and IL-35-secreting DCs in CIA. For that, we immunized the CIA-susceptible CD11b^−/−^ mice with collagen II (CII) in CFA. When over 80% of mice were already showing at least mild scores of the disease (footpad redness and/or swelling of one limb), we transferred a total of 5 × 10^6^ tolDCs/mouse via i.p. injection. The treatment was repeated 2 days later, and the progression of the disease was then scored for 1 extra week (Figures 4A,B). The combination of the two tolDC lines increased the chances of scores being reduced (Figures 4C,D). Moreover, in the group of mice treated with the combination of IL10-DCs + IL35-DCs, 75% of mice showed capacity for an intermediate recovery (over 1.5 points in score from the day of the second DC injection to 1 week later), while the groups that were treated with ctrl-DCs, IL10-DCs, or IL35-DCs alone could also reach reduced rates for the same criteria (25, 50, and 33.33%, respectively). Although no significant differences were observed in the linear phase of CII-specific IgG2a or IgG1 antibodies dilution in the sera of mice 1 week after treatment, the group that was injected with IL10-DC + IL35-DCs showed a tendency to produce reduced CII-IgG1 levels, demonstrated by a lower ratio of antibody concentration between the sera of DC-injected mice and of non-injected mice (Supplementary Figure 3). In another approach, we tested the systemic delivery of IL-10 in combination or not with IL-35 in the prevention of the disease's onset. For that, mice had a cytokine-secreting implant inserted subcutaneously in their back before the onset of the CIA (Figure 4E). Our results showed that the scores were kept lower in mice with IL-10- or IL-35-secreting implants. The systemic delivery of the two cytokines simultaneously, however, did not protect mice from the disease. In the mixed cytokine devices, the ratio of cytokine-producing cells was 1:1, but the total number of cells per device was the same as in the other groups, suggesting that the final concentration of both cytokines was probably suboptimal in the IL10 + IL35 implant group (Figures 4F,G). FoxP3^+^ Tregs, Bregs, iTr35, and Tr1 subpopulations in the spleen and inguinal lymph nodes of the mice from the implant groups and the mice that were treated with the DCs were assessed but no differences were found (data not shown). These results indicated that the systemic delivery of either IL-10 or IL-35 alone were significantly effective in the prevention of the disease onset, while the combination of the two cytokines did not show an advantage on it. However, it was only the combination of the IL-10 and IL-35 secreting tolDC lines that was more efficient at reducing the disease scores and promoting a robust recovery in mice with less severe forms of the disease.

### The Combination of IL10-DCs and IL35-DCs Did Not Alter Pre-established Memory Through Treg Expansion

**Figure 5:**
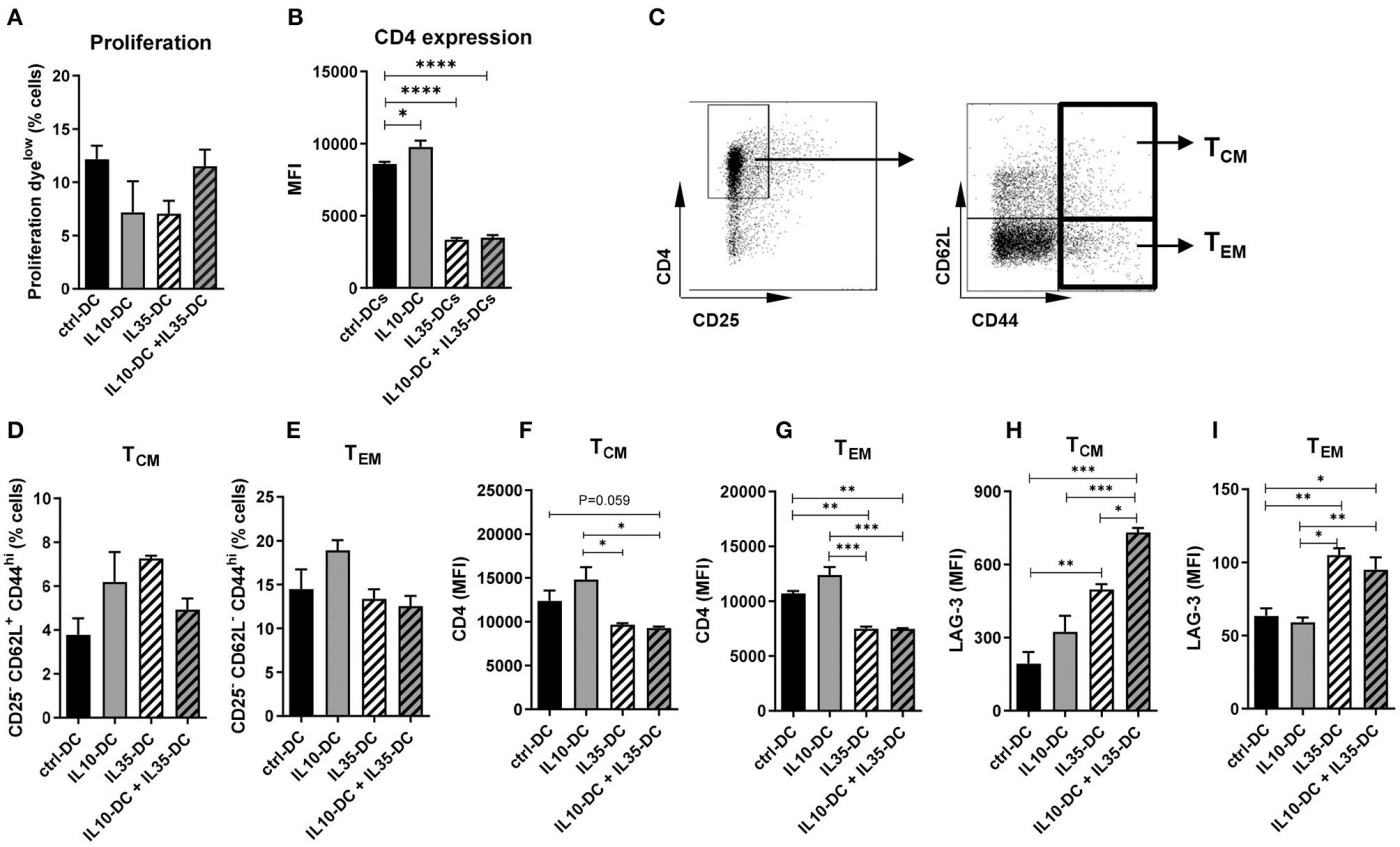
The combination of IL10-DC and IL35-DC induced upregulation of LAG-3 expression on memory CD4^+^ T cells. CD4^+^ T cells were isolated from the spleens of OVA-immunized mice, labeled with a proliferation dye, and put in co-culture with OVA-pulsed DCs for 3 days. Flow cytometric analysis of **(A)** T cell proliferation after co-cultures and **(B)** CD4 expression of total cells. **(C)** Gating strategy to determine T central memory (T_CM_–CD62L^+^CD44^high^–**D**) or T effector memory (T_EM_–CD62L^−^CD44^high^–**E**) cells. CD4 expression on **(F)** T_CM_ and **(G)** on T_EM_, and LAG-3 expression on **(H)** T_CM_ and **(I)** on T_EM_ (given in MFI values). Data are representative of at least three independent experiments with T cells isolated from 3 to 5 independently immunized mice (*n* = 3–5, each experiment). *P < 0.05; **P < 0.005, ***P < 0.001, ****P < 0.0001.

As our data indicated that the effect of the tolerogenic cell lines combined was in reducing pre-established memory activation, we generated memory T cells by immunizing mice against OVA (0.5 mg/mL) in CFA to further investigate it. Four weeks after the initial injection, CD4^+^ T cells were extracted from spleens and inguinal lymph nodes, labeled with a proliferation dye and co-cultured with OVA-pulsed tolDCs for 3 days. All DC lines induced comparable CD4^+^ T cell proliferation (Figure 5A) but CD4 MFI of total T cells was found reduced after the co-culture with IL35-DCs alone or in combination with IL10-DCs (Figure 5B). When central memory cells (T_CM_, CD25^−^ CD62L^+^ CD44^high^) and effector memory cells (T_EM_, CD25^−^ CD62L^−^ CD44^high^) compartments were investigated, no differences in the percentage of central and effector memory cells were observed between the experimental groups (Figures 5C–E). CD4 expression was downregulated in T cells primed by IL35-DCs alone or in combination with IL10-DCs was found in both, T_CM_ and T_EM_ compartments (Figures 5F,G).

### The Combination of IL10-DCs and IL35-DCs Induced High LAG-3 Expression in T_CM_

IL-35 was reported to induce LAG-3, PD-1, and TIM-3 on intratumoral CD4^+^ and CD8^+^ T cells (25). LAG-3 has been recently shown to selectively bind to stable peptide-MHC class II complexes, regulating CD4^+^ T cell activation in an expression-level-dependent fashion (26), therefore we tested if LAG-3 expression was altered by the IL35-DCs and what consequences the association of the IL35-DC and IL10-DC lines would bring to T cell fate. Indeed, LAG-3 expression was upregulated in both, central and effector memory compartments, when T cells had been co-cultured with IL35-DCs (Figures 5H,I). In addition to that, when IL35-DCs were combined with IL10-DCs, the LAG-3 MFI in the T_CM_ compartment was even more increased (Figure 5H). We also tested TIM-3 but there were no changes in its expression among the subgroups tested (data not shown).

**Figure 6:**
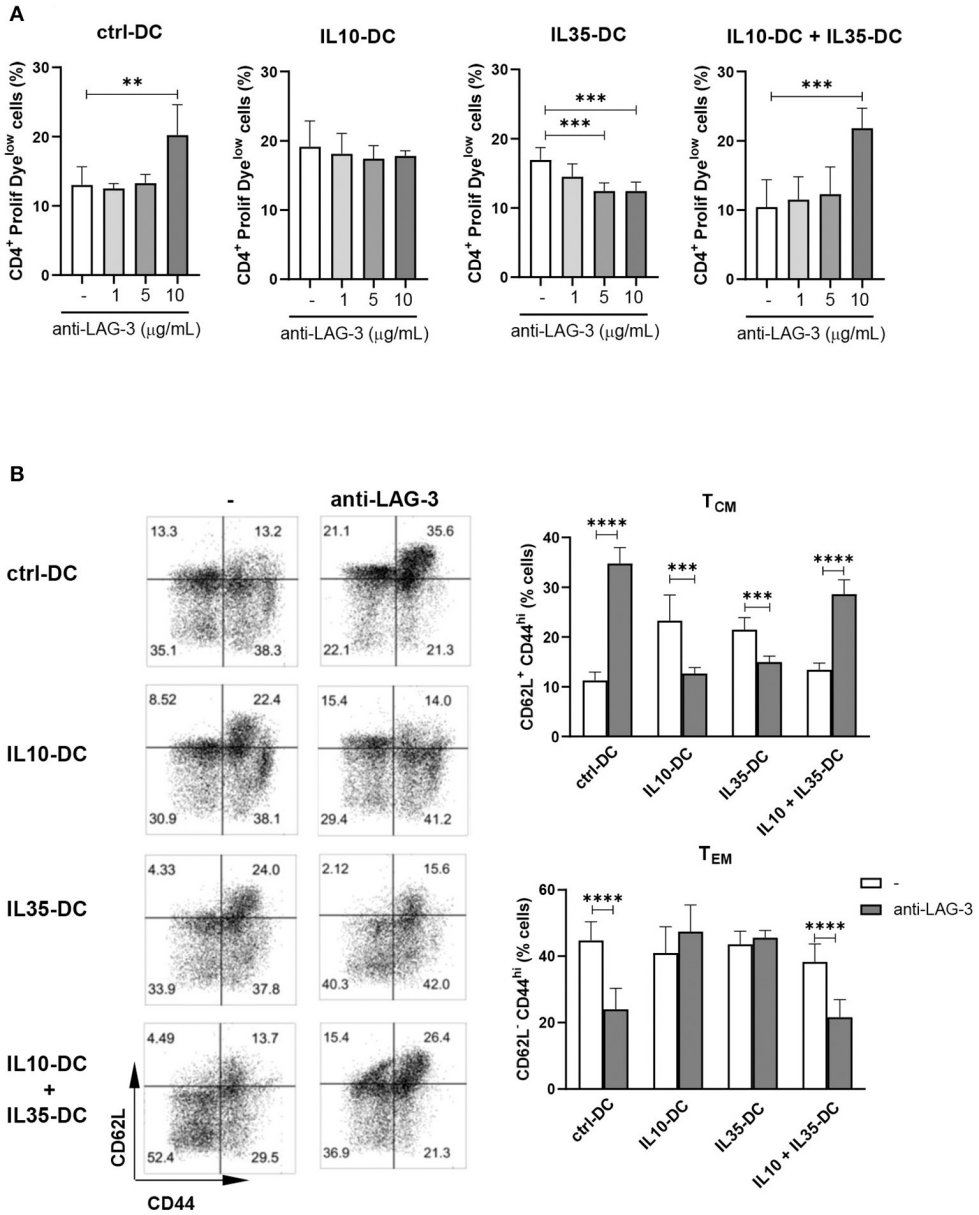
IL10-DCs in combination with IL35-DCs arrest memory expansion through LAG-3. CD4^+^ T cells were isolated from the spleens of OVA-immunized mice, labeled with a proliferation dye, and put in co-culture with OVA-pulsed DCs in the presence of anti-LAG-3 blocking antibody for 3 day. Flow cytometric analysis of **(A)** T cell proliferation after co-cultures and **(B)** CD4^+^ memory (CD62L^+^CD44^high^) or effector (CD62L^−^CD44^high^) cell population. Data are representative of at least two independent experiments with T cells isolated from five independently immunized mice (*n* = 5, each experiment). **P < 0.005, ***P < 0.001, ****P < 0.0001.

When the co-cultures were performed in the presence of LAG-3 blocking antibody, CD4^+^ T cell proliferation induced by ctrl-DCs and by the combination of IL10-DCs plus IL35-DCs was found increased. When T cells were primed by IL35-DCs, on the contrary, proliferation was reduced in a dose-dependent manner (Figure 6A). The effect of the blockade of LAG-3 was mostly observed in the reactivated T_CM_ compartment: in the presence of the antibody we observed an expansion of memory T cells after co-cultures with ctrl-DCs or with the combination of IL10-DC + IL35-DCs, and a reduction when co-cultures were done with IL10-DCs or IL35-DCs. In the T_EM_ compartment, the effect observed was the opposite: the blockade of LAG-3 decreased the expansion of the effector memory T cells in the first two groups mentioned above (Figure 6B). Altogether these results indicated that the combination of IL10-DCs and IL35-DCs reduced T cell activation through the downregulation of CD4 and upregulation of LAG-3 in the re-activated central memory cells, preventing them to be expanded, thus arresting excessive response during an inflammatory scenario.

## Discussion

DC vaccination relies on the manipulation of DCs to influence the immune system in an antigen-specific manner toward a tolerogenic or an immunogenic response. The study of DC biology is, however, limited by the scarcity and instability of DCs and thereby, the MutuDC lines represent a great tool to define the benefits of different immunomodulatory molecules in DC-based therapy. For this study, we employed our MutuDCs and generated a new tolerogenic DC line that constitutively produced high levels of IL-10.

TolDCs are characterized by stable low expression of co-stimulatory and MHC molecules, and altered cytokine secretion, leading T cells to anergy or promoting regulatory T cell differentiation. In addition to that, IL-10 is a major player in the anti-inflammatory response, classically known to inhibit T cell responses due to its modulatory effects on APCs or acting directly on the T cells [reviewed by Saraiva et al. (22)]. Moreover, IL-10 is also involved in the differentiation of Tr1 cells, which are able to suppress antigen-specific responses, thus having an important effect on the induction and maintenance of the peripheral tolerance (27). The IL10-DC line displayed many aspects of tolDCs, probably due to the constant and robust exposure to the tolerogenic cytokine secreted. Additionally, our results showed their ability to induce Tr1 differentiation and restrain *in vivo* antigen-specific inflammation. Still, they were able to induce substantial T cell response both *in vitro* and *in vivo*, indicating an advantage over conventional cytokine therapy.

The IL35-DC line was shown to induce suppressive T cells and efficiently reduce EAE scores even after the Th1/Th17 balance was established (10). Our results presented in this study confirmed the tolerogenic properties of the IL-35 secreting MutuDCs. Furthermore, here we show that the combination of IL35-DCs and IL10-DCs promoted a robust recovery of sick mice in an autoimmune model of RA. RA is a chronic, inflammatory, systemic autoimmune disease that can severely damage the joints and impair life quality. There is no cure for RA and the progression of the disease can lead to irreversible disability. Even though it is generally difficult to cure an established autoimmunity, different treatments for RA that allow many to live a near-normal life are available. In fact, most of the drugs currently used to treat RA are anti-inflammatory immune modulators, like Infliximab (anti-TNFα), Abatacept (CTLA-4Ig), Tocilizumab (anti-IL6R), Rituximab (anti-CD20), Anakinra (IL1-R antagonist), Canakinumab (anti-IL1), and Tofacitinib (JAK inhibitor). Some of these approaches are also proposed to treat other autoimmune diseases, such as Crohn's disease, ulcerative colitis, psoriasis, and have been shown to help in most cases, but the constant need of monitoring the disease and adjusting the treatment accordingly makes it challenging to achieve clinical remission (28, 29). On top of this, those broad spectra acting drugs, present a strong impact on the host defense as they modulate inflammatory and immune mediators, creating an eminent risk of infection. Thus, targeting the pathogenic autoreactive cells and/or antibodies in an antigen-specific fashion can exempt the protective immune cells and healthy tissues from collateral damage. While peptide-MHC-specific monoclonal antibodies have not yet been approved for therapeutic use, tolDC-based immunotherapeutic approaches have been conducted in the past decades aiming to induce, enhance, or restore tolerance in an antigen-specific fashion. In fact, many clinical trials with DCs differentiated in the presence of tolerogenic factors, such as IL-10, vitamin D, NF-κB inhibitor, have been reported with positive results, but the establishment of a standard DC manufacturing protocol, their tolerogenic characteristics stability, mode of action, and so on still remain elusive (7). Cytokine therapy can also represent an alternative to treat autoimmunity. The administration of IL-10, for example, has already shown promising results in the treatment of RA (30) and psoriasis (31). Moreover, the association of IL-10 with other anti-inflammatory cytokines could also be advantageous. The cooperation of IL-10 and TGF-β in downregulating immune responses is widely known as their production and action are interrelated. Additionally, the two cytokines were shown to cooperate in inducing secondary hyporesponsiveness to alloantigen (32), generating potent regulatory cells (12), and even inhibiting humoral immune response in a synergistic fashion (33), which suggests that combinatory cytokine therapies could increase the efficacy of the treatment without the possible side effects caused by high doses of single mediators. Thus, our results with systemic delivery of anti-inflammatory cytokines also provide new insights on therapeutic strategies for systemic inflammatory diseases.

In the immunological synapse, CD4 acts as a co-receptor for MHC class II, contributing to the assembly of the TCR-MHC II complexes. CD4 expression was reported to be upregulated after TCR triggering, which in turn correlates with increased T cell proliferation (34). On the other hand, low expression of CD4 could decrease T cell sensitivity to antigens and the efficiency of TCR-peptide-MHC II (pMHCII) binding. Through the blockade of IL-35 or Treg-restricted deletion of IL-35, Turnis et al. showed that IL-35 was implicated in promoting the expression of the inhibitory receptors PD-1, TIM-3, and LAG-3 in CD8^+^ tumor infiltrating T cells (25). LAG-3 structurally resembles CD4 but binds to MHC class II with a higher affinity than CD4, acting as a regulator of the immune response. This receptor has been also reported to be expressed in FoxP3^+^ Tregs and Tr1 cells, but most of all, its expression correlates with high IL-10 secretion [reviewed by Anderson et al. (35)]. Using multiple *in vivo* systems to induce immune responses in LAG-3^−/−^ mice, Workman et al. showed that LAG-3 negatively regulated primary T cell expansion and memory development (36). Among memory T cells, the T_CM_ cells comprise a population of lymph node-homing and circulatory cells that have a greater capacity of proliferation upon reactivation; they have less co-stimulation dependency and have a lower activation threshold, thus they are more likely to become activated during a second encounter with the antigen and providing stronger and faster responses (37). The distinct effect observed on T_CM_ expansion in the presence of anti-LAG-3 blocking antibody indicated that the two cytokine-secreting cell lines work differently in the induction of tolerance, and that when combined, the effect observed is most likely the result of a cooperation between them. As shown by Maruhashi et al. (26), the inhibition of CD4^+^ T cell activation through LAG-3 is dependent on stable pMHCII recognition. Due to the impaired T cell priming activity of IL35-DCs, we find unlikely that stable pMHCII complexes were formed by these cells. IL10-DCs could, however, provide those stable complexes, thus preferentially leading to their binding to LAG-3 instead of CD4. Thus, we hypothesize that when the two cell lines were combined, LAG-3 expression in T cells was induced and potentiated by the combination of the two cytokines secreted, but the T cell-DC binding mainly occurred with IL10-DCs. This could explain the enhanced upregulation of LAG-3, but similar proliferation induced by the two tolDC lines combined. In this scenario, IL35-DCs alone would induce tolerance through a different mechanism other than LAG-3 expression, and T cell priming by IL10-DCs alone would not be affected by the blockade of LAG-3. Nevertheless, the decreased proliferation induced by IL35-DCs under anti-LAG3 blockade still requires further studies to investigate if IL-35 could prompt an inverse agonist activity of the blocking antibody in memory T cells.

Taken together, our results demonstrate that MutuDC lines represent a great tool to investigate the benefits of immunomodulatory molecules in the antigen-presentation context and thus could help to characterize and optimize potential treatments for autoimmune disorders where overly inflammatory conditions are established.

## Data Availability Statement

The raw data supporting the conclusions of this article will be made available by the authors, without undue reservation.

## Ethics Statement

The animal study was reviewed and approved by Cantonal Veterinary Office of Vaud, Switzerland.

## Author Contributions

MK, AE, MP, CL, MS, and VL conducted the experiments. MK, BS, and HA-O designed the experiments. MK wrote the paper. All authors contributed to the article and approved the submitted version.

## Conflict of Interest

The authors declare that the research was conducted in the absence of any commercial or financial relationships that could be construed as a potential conflict of interest.

## Supplementary Material

Supplementary Figure 1 Phenotypical characteristics of the IL10-DC line after transduction. Cells were analyzed for the indicated surface markers by Flow cytometry after lentiviral transduction. Data are representative of three independent experiments.

Supplementary Figure 2 IL10-DC induce antigen-specific T cell proliferation. 5 × 10^3^ transduced or untransduced DCs were seeded in 96-well plates and pulsed with OVA_257−264_ peptide (SIINFEKL), OVA_323−339_ peptide (OT-II peptide), or full-length ovalbumin (100 μg/mL) for at least 2 h before adding 5 × 10^4^ of naïve proliferation dye-labeled OT-I CD8^+^ or OT-II CD4^+^ T cells. Cells were left in co-culture with peptide-pulsed DCs for 3 days or with OVA-pulsed DCs for 4 days. Flow cytometric analysis of **(A)** CD8^+^ T cell proliferation, **(B)** perforin, and **(C)** granzyme B expression on CD8^+^ T cells; **(D,E)** CD4^+^ T cell proliferation. Data are representative of at least three independent experiments. Bar graphs indicate the mean of technical replicates in one representative experiment.

Supplementary Figure 3 The combination of IL10-DC and IL35-DC lines reduced the IgG1-CII antibody levels in CIA sick mice. Blood serum samples from sick mice were obtained after 28 days of CIA induction. **(A)** Collagen II-specific IgG1 and IgG2a antibodies. **(B)** Ratio between the average concentration of CII-specific antibodies in DC-injected mice and non-injected mice. Data are representative of two independent experiments (*n* = 3–8 mice/group/experiment).
