# Supplementary figures and images for: IL10- and IL35-Secreting MutuDC Lines Act in Cooperation to Inhibit Memory T Cell Activation Through LAG-3 Expression
Source: Front Immunol. 2021 Feb 17;12:607315. doi: 10.3389/fimmu.2021.607315 (PMC7925845; doi:10.3389/fimmu.2021.607315)

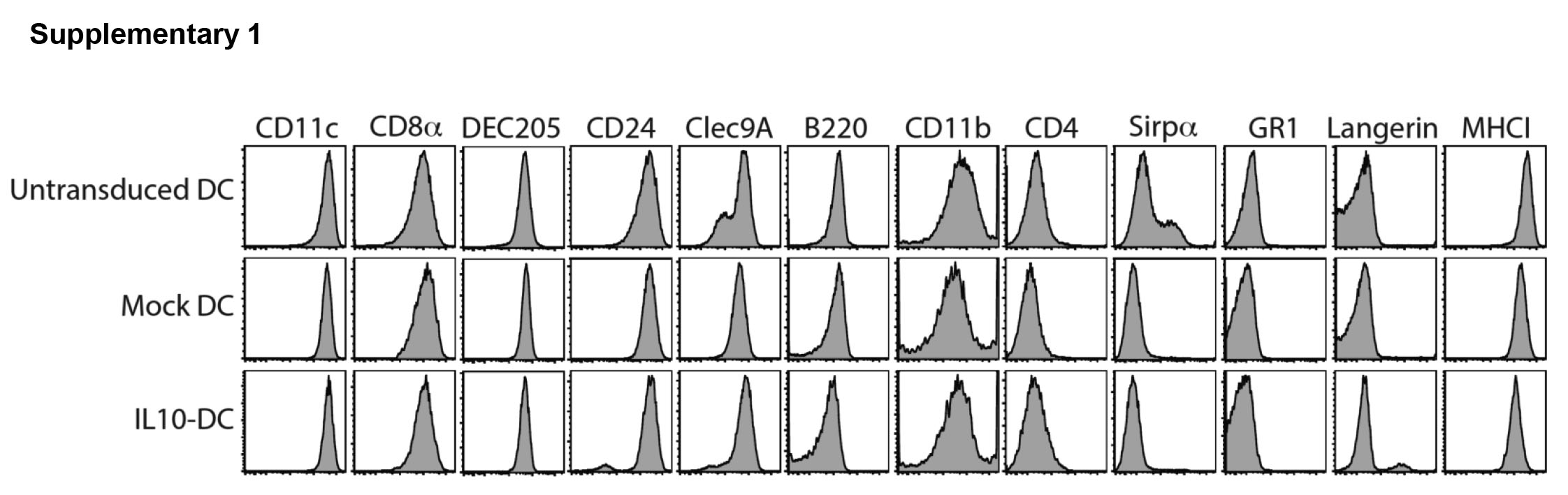

Supplement: Supplementary Figure 1 — Phenotypical characteristics of the IL10-DC line after transduction. Cells were analyzed for the indicated surface markers by Flow cytometry after lentiviral transduction. Data are representative of three independent experiments. [file Image_1.JPEG]

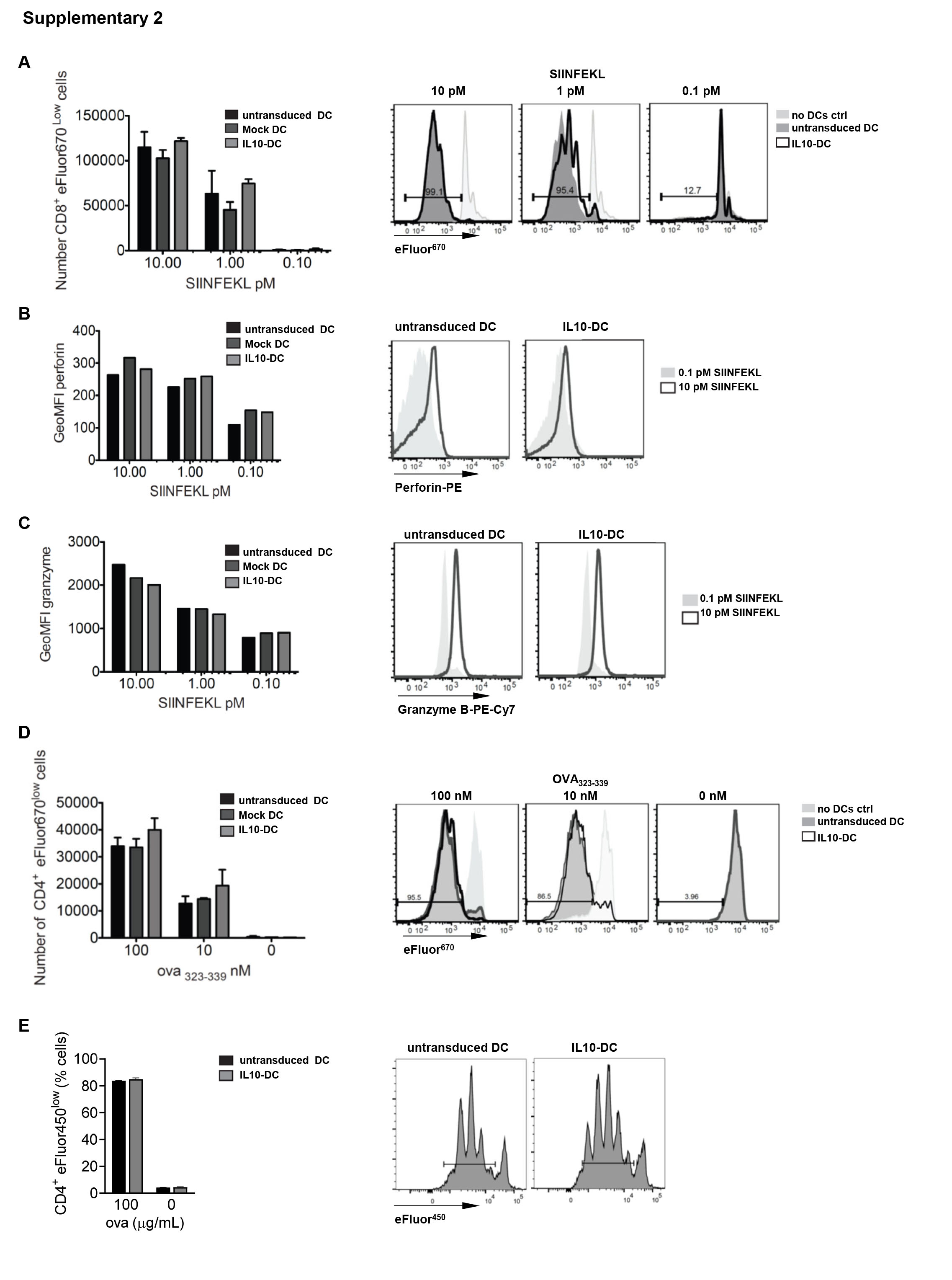

Supplement: Supplementary Figure 2 — IL10-DC induce antigen-specific T cell proliferation. 5 × 10^3^ transduced or untransduced DCs were seeded in 96-well plates and pulsed with OVA257−264 peptide (SIINFEKL), OVA323−339 peptide (OT-II peptide), or full-length ovalbumin (100 μg/mL) for at least 2 h before adding 5 × 10^4^ of naïve proliferation dye-labeled OT-I CD8+ or OT-II CD4+ T cells. Cells were left in co-culture with peptide-pulsed DCs for 3 days or with OVA-pulsed DCs for 4 days. Flow cytometric analysis of (A) CD8+ T cell proliferation, (B) perforin, and (C) granzyme B expression on CD8+ T cells; (D,E) CD4+ T cell proliferation. Data are representative of at least three independent experiments. Bar graphs indicate the mean of technical replicates in one representative experiment. [file Image_2.jpeg]

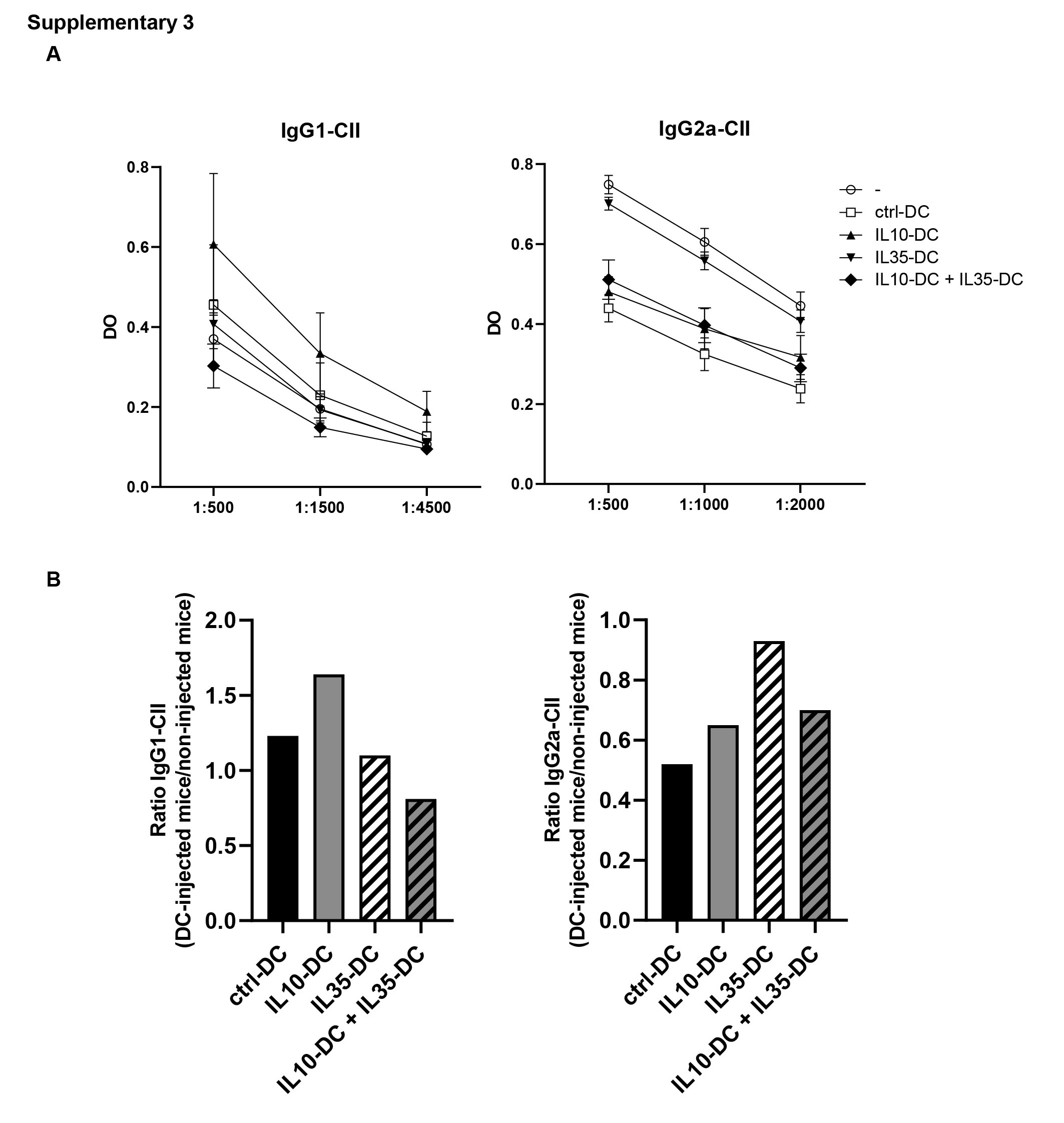

Supplement: Supplementary Figure 3 — The combination of IL10-DC and IL35-DC lines reduced the IgG1-CII antibody levels in CIA sick mice. Blood serum samples from sick mice were obtained after 28 days of CIA induction. (A) Collagen II-specific IgG1 and IgG2a antibodies. (B) Ratio between the average concentration of CII-specific antibodies in DC-injected mice and non-injected mice. Data are representative of two independent experiments (n = 3–8 mice/group/experiment). [file Image_3.jpeg]
